# Editorial: Targeting inflammation to prevent complications in stroke

**DOI:** 10.3389/fphar.2023.1229303

**Published:** 2023-06-13

**Authors:** Lourdes M. Varela, Elena Meseguer, Almudena Ortega-Gómez

**Affiliations:** ^1^ Instituto de Biomedicina de Sevilla (IBiS), Hospital Universitario Virgen del Rocío/ CSIC/ Universidad de Sevilla, Sevilla, Spain; ^2^ Dpto. Fisiología Médica y Biofísica, Facultad de Medicina, Universidad de Sevilla, Sevilla, Spain; ^3^ Department of Neurology and Stroke Center, APHP Bichat Hospital, France Université de Paris Cité, LVTS, Inserm U1148, Paris, France; ^4^ Department of Endocrinology and Nutrition, Virgen de la Victoria University Hospital, Málaga, Spain; ^5^ Biomedical Research Institute of Malaga (IBIMA-Bionand), Málaga, Spain; ^6^ Centro de Investigación Biomédica en Red de la Fisiopatología de la Obesidad y Nutrición (CIBEROBN CB06/003), Instituto de Salud Carlos III, Madrid, Spain

**Keywords:** stroke, hemorrhage, biomarker, heart failure, inflammation, neutrophils

This Research Topic, “*Targeting inflammation to prevent complications in stroke*”, focuses on the inflammatory response after stroke and other inflammatory diseases at the onset to prevent secondary injuries that result in poor outcomes. Our aim was to collect the latest findings of both basic and clinical research to contribute to the search for new biomarkers and therapies that help clinicians to assist patients in preventing and managing the appearance of complications during hospitalization. This Research Topic comprises two review articles and three research articles.

The World Stroke Organization has estimated that there are 12.2 million new strokes per year and its prevalence is increasing due to the aging of the population. Disruption of the blood-brain barrier (BBB) plays an important role in the development of neurological dysfunction in ischemic stroke. Neuroinflammation following ischemic stroke has been shown to disrupt the BBB, facilitating the passage of blood components into the perivascular space that results in neuronal cell death. Intracranial atherosclerotic stenosis (ICAS) is one of the most common causes of stroke worldwide and confers one of the highest risks of recurrent stroke compared to other causes of stroke. The study by Li et al. suggests that circular RNA hsa_circ_0003574 can be used as a biomarker to predict and diagnose ischemic stroke caused by ICAS. This circular RNA can aid in early detection and treatment of the condition, potentially improving patient outcomes. The breakdown of the BBB is also a characteristic that occurs after an aneurysmal subarachnoid hemorrhage (aSAH), a subtype of stroke that occurs primarily due to the rupture of a cerebral aneurysm. BBB injury leads to endothelial upregulation of adhesion molecules and infiltration of blood-derived cells, mainly neutrophils, through the compromised BBB. The study by Weng et al. found that the analysis of gene expression levels could highlight potential pathways and effector mechanisms linked to peripheral immune activation of neutrophils in aSAH, providing new insights into the development of immunoregulatory treatments for this disease. The study highlights the potential of neutrophil extracellular traps as a therapeutic target to reduce neuroinflammation involved in neuronal damage, BBB disruption, and microthrombi formation.

The other three studies published in this Research Topic aimed to evaluate different treatments to prevent complications after stroke. Oral anticoagulation (OAC) is commonly used to decrease the risk of stroke events and reduce all-cause death. However, intracranial hemorrhage (ICH) remains the most serious complication of OAC therapy. The systematic review and meta-analysis carried out by Zhou et al. evaluated the safety and efficacy of anticoagulant therapy in patients with atrial fibrillation who have a history of ICH. They analyzed data from seven cohort studies including 17.477 patients and two randomized controlled trials including 304 patients. The results showed that starting oral anticoagulation therapy reduced the risk of ischemic stroke/systemic embolism without increasing the risk of recurrent ICH, despite an increased risk of major bleeding. Compared to warfarin, direct oral anticoagulants (DOAC) had a lower risk of ischemic stroke/systemic embolism, recurrent ICH, and all-cause death. However, subgroup analyses revealed that the starting of OAC therapy was not necessarily beneficial for all populations, since this therapy had a higher risk of recurrent ICH than non-oral anticoagulation therapy in Asian populations.

Despite the decline in heart failure morbidity and mortality with current therapies, rehospitalization rates remain high, mainly due to disease-related ventricular remodeling. Current heart therapies, including angiotensin-converting enzyme inhibitors (ACEI) and angiotensin receptor blockers (ARB), show significant efficacy in reducing morbidity and mortality in patients with chronic systolic heart failure. However, in many cases, the progression of the disease continues unabated. The meta-analysis by Qin et al. suggests that, compared to ACEI and ARB, sacubitril-vasartan (ARNI) may be an effective and safe strategy to improve the left ventricular function and quality of life, and reduce readmission rate in patients with heart failure with mid-range ejection fraction (HFmrEF).

Finally, ferroptosis has been found to play a critical role in myocardial infarction, ischemia/reperfusion injury, and heart failure. Ferroptosis is a form of iron-dependent regulated cell death, and accumulated experimental data suggest that ferroptosis may be a novel target for various cardiovascular diseases. The study by Jiao et al. found that platelet-rich plasma can ameliorate lipopolysaccharide-induced ferroptosis and inflammation effects *in vivo* and *in vitro*, and could provide a new therapeutic direction to treat sepsis-induced myocardial dysfunction (SIMD) through the regulation of AKT/mTOR signaling pathways.

In conclusion, this Research Topic, “*Targeting inflammation to prevent complications in stroke*”, highlights various studies to develop potential targets or novel therapeutics for the diagnosis and treatment of cerebro-cardiovascular diseases.

